# Эволюция инсулинотерапии: прошлое, настоящее, будущее

**DOI:** 10.14341/probl13251

**Published:** 2024-01-24

**Authors:** Д. В. Куркин, Д. А. Бакулин, А. И. Робертус, Ю. А. Колосов, И. С. Крысанов, Е. И. Морковин, А. В. Стрыгин, Ю. В. Горбунова, И. Е. Макаренко, Р. В. Драй, Е. В. Макарова, Е. В. Павлова, Р. А. Кудрин, О. В. Иванова

**Affiliations:** Московский государственный медико-стоматологический университет им. А.И. Евдокимова; Волгоградский государственный медицинский университет; Московский государственный медико-стоматологический университет им. А.И. Евдокимова; Российский национальный исследовательский медицинский университет им. Н.И. Пирогова Ю.А.; Московский государственный медико-стоматологический университет им. А.И. Евдокимова; Московский государственный медико-стоматологический университет им. А.И. Евдокимова; Московский государственный медико-стоматологический университет им. А.И. Евдокимова; Волгоградский государственный медицинский университет; Московский государственный медико-стоматологический университет им. А.И. Евдокимова; ЗАО «Фарм-Холдинг»; ЗАО «Фарм-Холдинг»; Московский государственный медико-стоматологический университет им. А.И. Евдокимова; Университет Сантьяго де Компостела; Московский государственный медико-стоматологический университет им. А.И. Евдокимова; Волгоградский государственный медицинский университет; Московский государственный медико-стоматологический университет им. А.И. Евдокимова

**Keywords:** инсулин, открытие, сахарный диабет

## Abstract

В 2021 г. исполнилось 100 лет со дня открытия инсулина — события, навсегда изменившего жизнь людей с сахарным диабетом. Сегодня пациенты во всем мире ежедневно сталкиваются с чудом инсулинотерапии. Болезнь, от которой в 1920 г. дети и подростки умирали в течение 2 лет, превратилась в болезнь, которую можно контролировать и прожить с ней долгую продуктивную жизнь. За прошедший век великое открытие Бантинга, Беста и Коллипа навсегда изменило мир и спасло миллионы жизней. Данный обзор посвящен истории создания инсулина и его дальнейшего усовершенствования: от момента открытия до наших дней. Рассматриваются различные поколения инсулина: от животных до современных ультракоротких и базальных аналогов. Завершается статья кратким обзором современных направлений развития новых способов доставки и разработки новых молекул инсулина. За последнее столетие инсулинотерапия шагнула далеко вперед, что существенно улучшило качество жизни наших пациентов. Но исследования активно продолжаются, в том числе в области альтернативных способов доставки инсулина, которые являются более удобными для пациента, а также в области разработок «умных» молекул, которые будут обладать глюкозозависимым действием.

## Abstract (en)

2021 marks the 100th anniversary of the discovery of insulin, an event that forever changed the lives of people with diabetes mellitus. At present patients around the world experience the miracle of insulin therapy every day. A disease that used to kill children and teenagers in 2 years in 1920 has become a disease that can be controlled with a possibility to lead a long productive life. Over the past century, the great discovery of Banting, Best and Collip has forever changed the world and saved millions of lives. This review is devoted to the history of the development of insulin and its further improvement: from the moment of discovery to the present days. Various generations of insulin are considered: from animals to modern ultrashort and basal analogues. The article ends with a brief review of current trends in the development of new delivery methods and the development of new insulin molecules. Over the past century, insulin therapy has come a long way, which has significantly improved the quality of life of our patients. But research is actively continuing, including in the field of alternative methods of insulin delivery, which are more convenient for the patient, as well as in the development of «smart» molecules that will have a glucose-dependent effect.

## ВЕЛИКОЕ ОТКРЫТИЕ, ИЗМЕНИВШЕЕ МИР

В течение многих столетий развитие сахарного диабета (СД) приводило к значительному сокращению продолжительности жизни, а иногда и скорой мучительной смерти больного. Именно поэтому после открытия взаимосвязи между поджелудочной железой и повышением уровня глюкозы в крови многие ученые в начале XX в. предпринимали попытки лечения диабета экстрактами поджелудочной железы.

Необходимо отметить, что большое значение для выяснения роли поджелудочной железы в происхождении СД имели работы нашего соотечественника Леонида Васильевича Соболева, который в 1901 г. в своей ­диссертации опубликовал результаты экспериментальных исследований, подтверждающих роль островков Лангерганса в регуляции углеводного обмена [1].

В 1915 г. американский исследователь Исраэль Кляйнер обнаружил, что вытяжки из поджелудочной железы снижают уровень глюкозы у собак с удаленной поджелудочной железой, но не смог продолжить эту работу, поскольку в 1919 г. перешел в другой университет, где не было соответствующих условий для содержания лабораторных животных.

В 1916 г. румынский физиолог Николай Паулеско открыл, что вытяжка из поджелудочной железы снижает уровень глюкозы у собак после панкреаэктомии. Ученый смог возобновить работу только после окончания Первой мировой войны и в 1923 г. опубликовал сообщение, что инъекция произведенного им экстракта 24 марта 1922 г. снизила уровень глюкозы у пациента с диабетом.

Фредерик Бантинг не знал об этих открытиях, когда в 1920 г. начал свои поиски вещества, которое вскоре назовут инсулином. После окончания медицинского факультета Университета Торонто в 1917 г., службы в армии в качестве врача в канадской армии во время Первой мировой войны, а затем обучения на хирурга-ортопеда в детской больнице в Торонто в 1919 г. Бантинг открыл практику в Лондоне, Онтарио.

В 1920 г. Бантинг смог встретиться с профессором Дж. Дж. Маклеодом, который возглавлял кафедру физио­логии в Университете Торонто и считался в то время очень авторитетным специалистом по углеводному обмену. Во время их встречи Бантинг предложил реализовать свои идеи о перевязке протока поджелудочной железы в лаборатории Маклеода. Несмотря на скептицизм, Маклеод принял это предложение, и в мае 1921 г. Бантинг и Маклеод начали эти эксперименты. Поскольку им нужна была помощь в измерении уровня глюкозы, они наняли Чарльза Беста, выпускника четвертого курса факультета физиологии и биохимии.

Экспериментальная работа продолжалась в течение всего лета 1921 г., и в ноябре в Журнал лабораторной и клинической медицины (Journal of Laboratories and Clinical Medicine) были представлены результаты наблюдения за собаками, которым успешно проводили терапию вытяжкой из поджелудочной железы. В середине ноября Бантинг выдвинул гипотезу о том, что, возможно, проще приготовить экстракт из поджелудочной железы плода теленка до того, как в ней разовьются экзокринные клетки поджелудочной железы.

Бантинг и Бест извлекли и обработали несколько эмбриональных поджелудочных желез телят с бойни и успешно протестировали их экстракт на собаке с диабетом. Обнаружив, что их экстракт растворим в спирте и может быть выделен из легкодоступной поджелудочной железы взрослого крупного рогатого скота, они начали многодневное исследование с участием собаки с диабетом, известной как Марджори. Она прожила 70 дней.

В то же время испытания нового вещества на людях постоянно откладывались из-за постоянных проблем с очисткой препарата, которые смог решить Джеймс Коллип, профессор биохимии в Университете Альберта в Эдмонтоне, который по счастливой случайности находился в Торонто в творческом отпуске. Когда очищенный Коллипом препарат (носивший название сыворотки Маклеода) ввели Леонарду Томпсону, 13-летнему мальчику, уже к тому времени 2 года страдавшему СД 2 типа (СД2), глюкозурия и кетонурия полностью исчезли на 2 дня.

После первого успешного применения препарата работа исследователей получила широкую поддержку. Лаборатории Коннаутского университета в Торонто согласились на крупномасштабное производство экстракта поджелудочной железы и его дальнейшую оценку для клинического использования. В марте 1922 г. в журнале Канадской медицинской ассоциации уже был опубликован документ, описывающий первых 7 пациентов, успешно пролеченных экстрактом поджелудочной железы.

Инсулином новый препарат впервые назвал Маклеод, выступая 3 мая 1922 г. на конференции в Вашингтоне. В последующем Бантинг, Бест и Коллип передали патентные права на инсулин Университету Торонто за символический 1 доллар, после чего последовало создание комитета для управления патентами на инсулин, лицензирования производителей в Северной Америке и упрощения его производства в других странах.

Открытие Бантинга, Беста и Коллипа привлекло внимание Джорджа Клоуза — первого директора по исследованиям в компании Eli Lilly and Company (США).

Инсулин Бантинга и Беста имел продолжительность действия около 6 ч с высокой вариабельностью эффекта, его применение сопровождалось выраженными гипо- и гипергликемическими состояниями. Ученые стали разрабатывать способы получения более чистых препаратов инсулина.

К началу 1923 г. компания Lilly уже производила инсулин на постоянной основе, однако колебания в эффективности достигали 25% от партии к партии.

Более стабильный инсулин был получен главным химиком компании Lilly Джорджем Уолденом, который в декабре 1922 г. разработал метод изоэлектрического осаждения, который обеспечивал в десятки раз большую стабильность и чистоту субстанции.

В мае 1922 г., когда стало очевидно, что Connaught Labs не хватает технологий и мощностей для производства количества инсулина, достаточного для удовлетворения быстро растущего спроса на терапию, Клоуз подписал соглашение с Университетом Торонто, которое позволило Connaught Labs и Lilly сотрудничать в разработке методов крупномасштабного производства. Это позволило Connaught Labs поставлять весь инсулин, необходимый для нужд Канады, а Eli Lilly — стать первой компанией, производящей инсулин для США.

В 1922 г. датский физиолог и лауреат Нобелевской премии по физиологии Август Крог путешествовал по США в сопровождении своей жены Мари, у которой недавно был диагностирован диабет. Узнав об открытии, Мари убедила своего мужа связаться с Маклеодом и предложить добавить посещение Торонто к их маршруту. Во время своего визита Крог познакомился с Бантингом и посетил заводы по производству инсулина, а затем получил права на производство инсулина в Дании. Эта инициатива привела к созданию Nordisk Pharmaceuticals. Именно профессор Крог поспособствовал выдвижению работы Бантинга и Маклауда на Нобелевскую премию по физиологии и медицине, которая была присуждена 25 октября 1923 г. [2].

Впоследствии с помощью ряда исследований была выяснена биохимия инсулина и его рецептора. Стало известно, что инсулин представляет собой гетеродимерный пептид, состоящий из двух цепей — А (21 аминокислота) и В (30 аминокислот), которые появляются в гексамере, стабилизированном двумя Zn2+. Две цепи соединяются вместе посредством двух межпептидных дисульфидных связей (CysB7 с CysA7 и CysB19 с CysA20) и внутрипептидной дисульфидной связи (CysA6 с CysA11) [3].

В 1949 г. Сазерленд и соавт. выделили из поджелудочной железы пептид, который был назван глюкагоном, и тогда же стало известно, что это вещество вырабатывается также в островках поджелудочной железы, но другим типом клеток (a-клетками) после расщепления пре-проглюкагона до глюкагона под влиянием главным образом проконвертазы-2 (PC2). Позже стало известно также о δ-клетках, секретирующих соматостатин, и γ-клетках, вырабатывающих панкреатический полипептид. Паракринные взаимодействия играют важную роль в регуляции секреции глюкагона α-клетками. И инсулин, и соматостатин высвобождаются в условиях гипергликемии, и эти гормоны способны ингибировать секрецию глюкагона.

На рисунке 1 представлено взаимодействие между тремя основными типами островковых клеток.

**Figure:**
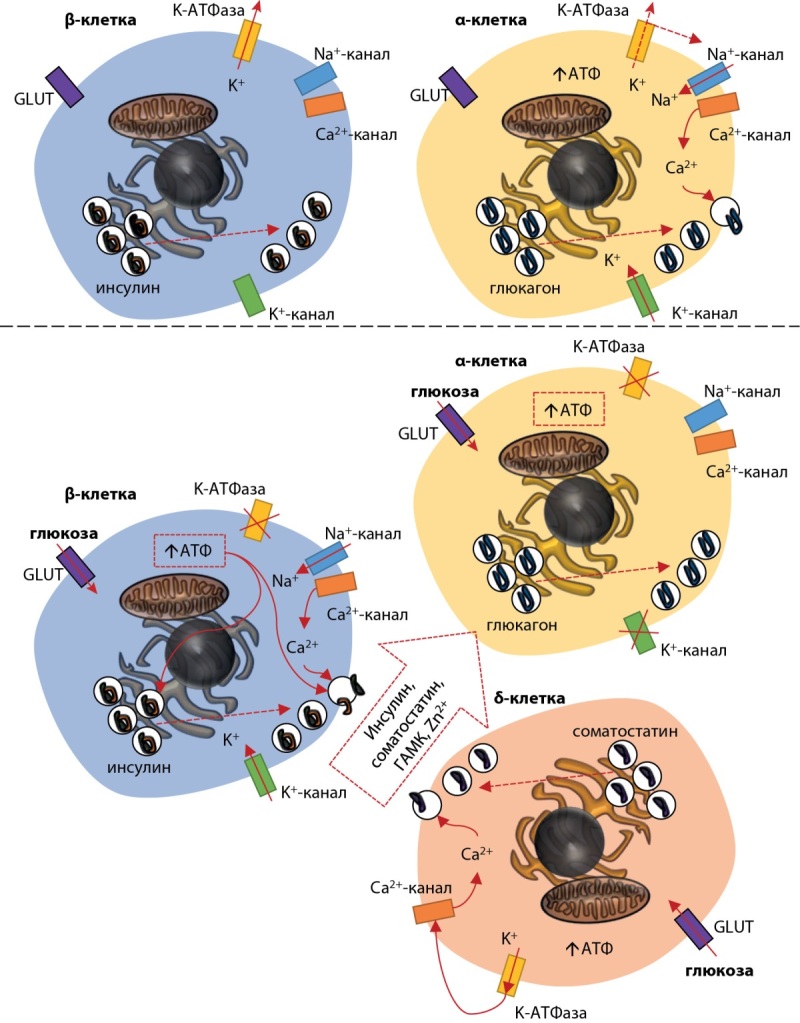
Рисунок 1. Паракринные взаимодействия β-, α- и δ-клеток островков поджелудочной железы.

## ЭРА ЖИВОТНЫХ ИНСУЛИНОВ: СТАНДАРТИЗАЦИЯ, ОЧИСТКА И ПРОТАМИН РЫБ

Вскоре после того, как новость об открытии инсулина распространилась по всему миру, возникла острая необходимость в расширении масштабов его производства. В дальнейшем Джордж Уолден (Eli Lilly and Co) существенно улучшил чистоту и стабильность инсулина; стандартизация достигалась изоэлектрическим осаждением и перекристаллизацией [4].

Стандартизация активности разных партий достигалась внедрением биологических анализов оценки сахароснижающего действия, в результате чего на каждом флаконе инсулина было указано количество содержащихся в нем сахароснижающих единиц. Мировое сообщество приняло определение международной единицы инсулина, данное Университетом Торонто, основанное на степени снижения уровня глюкозы, достигаемой у стандартного 2-килограммового голодного кролика (1 ЕД должна снизить глюкозу крови у животного на 2,5 ммоль/л). Дозирование инсулина и в настоящее время осуществляется по международным единицам (1 ЕД эквивалентна 34,7 мкг чистого кристаллизованного человеческого инсулина). Концентрации инсулина в различных препаратах менялись на протяжении многих лет, но согласованные усилия во всем мире привели к принятию стандартов для повышения безопасности его применения.

К 1930 г. Инсулин Лео (торговое наименование) был доступен в 28 странах.

При поддержке комитета по инсулину Университета Торонто методы выделения и очистки инсулина из поджелудочной железы телят, а затем свиней быстро распространились по всему миру. Среди производителей были Connaught Laboratories в Канаде, Eli Lilly и другие фармацевтические компании в США, Nordisk, а затем Novo в Скандинавии, Совет медицинских исследований в Великобритании, Hoechst в Германии.

Изначально продолжительность действия обычного инсулина (также известного как «растворимый», или инсулин «Торонто») составляла всего 4–6 ч, поэтому его вводили несколько раз в день.

Разработка в Канаде в 1936 г. протамин-цинкового инсулина положила начало эре базальной инсулинотерапии [5]. Хагедорну и Дженсену удалось скомбинировать инсулин с протамином рыб, который, кристаллизуясь с гексамерами инсулина, задерживает высвобождение его активных мономеров в кровоток. В том же году Скотт и Фишер предложили добавить цинк к инсулину для создания протамин-цинково-инсулинового комплекса.

Только в 1946 г. был внедрен нейтральный протаминовый инсулин Хагедорна (НПХ) с длительностью действия до 4–12 ч. Он обеспечивал более стабильные профили глюкозы и мог применяться в сочетании с инсулином короткого действия. Попытки создать инсулины более длительного действия, такие как суспензии инсулина ленте (семиленте, ленте и ультраленте), в 1950-х годах не были успешны [6].

Первый инсулин НПХ был произведен и продан компанией Nordisk в 1950 г. Продолжительность действия была выше, но источником его получения оставались животные: коровы или свиньи, а протамин производили из спермы лосося, поэтому их применение часто сопровождали реакции гиперчувствительности. Иммуногенность инсулина вызывала образование антител и местные реакции, часто осложнявшиеся липоатрофией. В 1973 г. начали производить высокоочищенные инсулины (монокомпонентные). В процессе разработки высокоочищенных инсулинов концентрацию проинсулина (основного иммуногенного агента) удалось снизить более чем в 50 000 раз, это позволило минимизировать развитие антител к инсулину, что привело к пролонгации его активности и, соответственно, к повышению общей эффективности его применения.

Основные вехи истории создания и усовершенствования препаратов инсулина представлены на рисунке 2.

**Figure:**
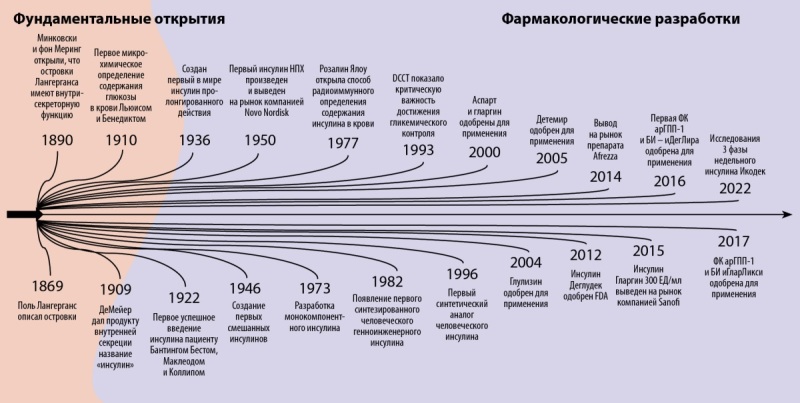
Рисунок 2. Эволюция инсулинотерапии: от Лангерганса до наших дней [38].

## ЧЕЛОВЕЧЕСКИЕ ИНСУЛИНЫ: СОЛО КИШЕЧНОЙ ПАЛОЧКИ

Молекулярную структуру инсулина расшифровывали постепенно, и в 1955 г. его аминокислотная последовательность была описана Ф. Сэнгером, а в 1967 г. Р. Шанс определил структуру проинсулина. Трехмерную структуру инсулина двумя годами позже описал D. Crowfoot-Hodgkin.

Исследования инсулина привели к установлению нескольких ключевых фактов — инсулин существует в виде мономеров и димеров с естественной тенденцией к агрегации в гексамеры, определено точное положение трех дисульфидных связей в структуре проинсулина (и положение двух связей в обработанном инсулине), установлены N- и С-концевые участки цепи А, гидрофобные остатки В-цепи в совокупности, это позволило не только к лучше понимать физиологию и фармакологию, но и значительно приблизило зарю генной инженерии и синтез человеческого инсулина.

Спустя полвека после открытия в 1978 г. методами генной инженерии по технологии рекомбинантной ДНК в E. coli, которая была создана двумя годами ранее для производства соматостатина, был произведен первый человеческий полусинтетический инсулин.

Дэвид Гёддель и его коллеги из Genentech синтезировали первую рекомбинантную ДНК (рДНК) человеческого инсулина объединением цепей А- и В-инсулина посредством экспрессии генов в E. coli.

Рекомбинантный человеческий инсулин был выпущен на рынок в 1983 г. компанией Eli Lilly and Company под названием Humulin. За ними последовали и другие производители (Novo Nordisk и Hoechst).

Рекомбинантный человеческий инсулин был полностью гомологичен гормону человека, имел низкий риск иммунного ответа, а также был значительно дешевле в производстве.

Чтобы воспроизвести физиологическую прандиальную секрецию инсулина, идеальный инсулин должен быстро всасываться в кровоток из подкожно-жировой клетчатки.

Первоначально производившиеся в виде очень разбавленных растворов, содержащих 10 международных единиц на 100 миллилитров (или 10 ЕД/мл), коммерческие препараты инсулина стали более концентрированными к концу 1970-х годов — концентрация инсулина выросла с 20 до 40 ЕД/мл, а затем и до 100 ЕД/мл. Осознание того, что людям с тяжелой резистентностью к инсулину необходимо вводить очень большие объемы препаратов, способствовало разработке ультраконцентрированных форм инсулина.

Концентрирование человеческого инсулина обычно приводит к пролонгации его действия. Поэтому введение одинакового количества международных единиц одного и того же препарата разной концентрации вызывает неодинаковые по выраженности фармакодинамические и, соответственно, клинические эффекты. Так, ультраконцентрированный человеческий инсулин (например, обычный человеческий инсулин 500 ЕД/мл) имеет фармакокинетический профиль, промежуточный между профилями 100 ЕД/мл и НПХ.

Существенные изменения претерпели и инструменты для введения инсулина: от больших многоразовых стеклянных шприцев и игл, которые было необходимо стерилизовать перед использованием, к одноразовым пластиковым шприцам, шприцам-ручкам, инфузионным системам и практически безболезненным инъекциям.

Несмотря на то что производство инсулинов НПХ было первой и достаточно успешной попыткой увеличить продолжительность действия, сегодня инсулиновые препараты данной группы значительно уступают современным синтетическим аналогам по показателям вариабельности гликемии. Кроме того, фармакокинетический профиль инсулинов НПХ зависит от вводимой дозы, что обуславливает риск гипо- или гипергликемии.

Основной целью разработки аналогов инсулина является преодоление вышеописанных проблем. Аналоги инсулина получают путем изменения аминокислотной последовательности для достижения следующих целей: модификация фармакокинетики, имитация физиологической секреции инсулина, управление гликемией натощак и после приема пищи, минимизация риска гипогликемии.

## СОВРЕМЕННЫЕ АНАЛОГИ ИНСУЛИНА: СВЕТЛОЕ НАСТОЯЩЕЕ ИНСУЛИНОТЕРАПИИ

## Быстродействующие аналоги инсулина

Инсулин лизпро

Первый быстродействующий аналог инсулина — инсулин лизпро был разработан в 1996 г. Ричардом Ди Марчи и его исследовательской группой в лабораториях Lilly. Ученые заметили, что инсулиноподобный фактор роста-1 структурно подобен инсулину, образует гексамеры с меньшей силой сцепления, что позволяет более быстро диссоциировать на мономеры. Они установили, что это связано с разницей в аминокислотной последовательности в С-концевых положениях 28 и 29 В-цепи между IGF-1 (Lys-Pro) и инсулином (Pro-Lys). Затем оказалось, что «простая» инверсия двух аминокислот обеспечила более быструю диссоциацию гексамеров инсулина после подкожной инъекции с более быстрым всасыванием в кровоток и меньшей продолжительностью действия. В клинических исследованиях было показано, что инсулин лизпро снижает постпрандиальную гликемию лучше, чем человеческий инсулин [7]. Лизпро начинает действовать приблизительно через 15 минут после подкожной инъекции и достигает максимальной концентрации в сыворотке (Cmax) через 30–60 минут. Продолжительность действия инсулина лизпро составляет 3–4 ч. Абсолютная биодоступность препарата может достигать 77% при дозах от 0,1 до 0,2 ЕД/кг. По эффективности снижения уровня гликемии инсулин лизпро эквивалентен человеческому инсулину.

Сравнительно недавно была выпущена модификация первого аналога инсулина — инсулин лизпро с ультрабыстрым действием. Ультрабыстродействующий лизпро содержит дополнительный цитрат для увеличения всасывания за счет повышения местной проницаемости сосудов и трепростинил для усиления локальной вазодилатации [8].

В исследованиях ультрабыстрый лизпро показал достижение концентрации половины максимальной дозы через 10,8 минут после инъекции, что на 6 минут быстрее, чем ультрабыстрый инсулин аспарт, и вызвал большее снижение постпрандиальной гликемии, чем у ультрабыстрого инсулина аспарт [9].

Увеличение концентрации ультрабыстрого инсулина лизпро до 200 Ед/мл не изменяет его фармакокинетический и фармакодинамический профили, что обеспечивает взаимозаменяемость с другими препаратами инсулина лизпро [10].

Инсулин аспарт

Аспарт — аналог прандиального инсулина короткого действия, одобренный и выпущенный на рынок в 2000 г., имеет фармакокинетический профиль, сравнимый с лизпро, включая его биоактивность, и образуется путем замены аминокислоты B28 пролина на аспарагиновую.

Эта модификация устраняет поверхность притяжения мономер-мономер и дополнительно усиливает отталкивание между заряженной аспарагиновой кислотой и соседней.

В результате гексамер инсулина быстрее диссоциирует до мономеров и начинает свое действие в течение 10–20 минут после подкожного введения, достигая максимальной концентрации в сыворотке через 40–50 минут после начала действия, которое продолжается в течение 3–5 ч.

Ультрабыстрый инсулин аспарт, выпущенный в 2017 г., содержит L-аргинин в качестве стабилизатора и ниацинамид для увеличения всасывания за счет усиления подкожного кровотока [11]. По сравнению с обычным инсулином аспарт эта лекарственная форма приводит к более раннему (на 5 минут) началу действия, быстрому развитию сахароснижающего эффекта (на 74% в первые полчаса после инъекции) и сокращению продолжительности действия на 14 минут [12].

Согласно данным регистрационных исследований, у пациентов с СД 1 типа (СД1) эти эффекты приводили к дополнительному снижению HbA1c на 0,08% и улучшению контроля постпрандиальной гликемии с меньшим количеством гипогликемических событий по сравнению с инсулином аспарт. В то же время подобные выводы могут быть необъективными, поскольку в рамках клинических исследований дозирование новых препаратов в фазе титрации дозы может осуществляться с большей осторожностью, чем в реальной клинической практике. Однако использование ультрабыстрого аспарта для непрерывной подкожной инфузии оказалось не таким успешным и не улучшало гликемический контроль по сравнению с традиционной формой [13][14].

Инсулин глулизин

Глулизин является еще одним аналогом прандиального инсулина короткого действия по сравнению с лизпро и аспартом, препарат появился на рынке только в 2004 г. [15].

Глулизин получают заменой двух аминокислот. Аспарагин в положении В3 заменен лизином, а лизин в положении В29 заменен глутаминовой кислотой. Эти изменения увеличивают скорость перехода гексамера в димеры и мономеры после растворения в подкожной ткани, а ­также снижают изоэлектрическую точку инсулина с 5,5 (нативный инсулин) до 5,1, что, существенно улучшает его растворимость. Глулизин начинает действовать быстро (в течение 20 минут после подкожного введения) и через 1 ч достигает Cmax. Действие препарата глулизин продолжается в течение 4 ч, а его абсолютная биодоступность после подкожного введения составляет примерно 70%.

В отличие от инсулинов лизпро и аспарт в инсулине глулизин в качестве вспомогательного вещества используется полисорбат 20 вместо Zn2+, что позволяет достичь более быстрого начала действия. Удаление цинка приводит к разрушению гексамера инсулина, что способствует абсорбции. Кроме того, полисорбат 20 представляет собой поверхностно-активное вещество, которое добавляют в качестве стабилизирующего агента, чтобы сбалансировать снижение стабильности из-за удаления цинка.

Систематический обзор и метаанализ сравнения прандиальных аналогов инсулина и нативного человеческого инсулина в отношении контроля уровня гликемии при СД1 показали, что прандиальные аналоги инсулина превосходят человеческий с точки зрения общего количества эпизодов гипогликемии, снижения постпрандиальной гликемии и общих результатов терапии [16][17].

## Базальные аналоги инсулина

Инсулин гларгин

В 2000 г. был одобрен первый аналог базального инсулина — гларгин, который навсегда изменил устоявшиеся представления о сложности и небезопасности инсулинотерапии.

Гларгин имеет два остатка L-аргинина, добавленные к С-концу В-цепи, и замену глицина на аспарагин. Эти модификации изменяют изоэлектрическую точку инсулина и предотвращают дезамидирующий эффект аспарагина, вызывая впоследствии более стабильную агрегацию, пролонгирующую высвобождение.

В результате осаждение происходит только при нейтральном рН (в месте инъекции), тогда как молекула растворяется при кислом рН (во флаконе). Инсулин гларгин имеет более благоприятный и плавный фармакодинамический профиль, чем инсулин НПХ, со средней продолжительностью действия 20 ч после однократного введения первой дозы, которая увеличивается при поддерживающей терапии. Переход с инсулина НПХ на инсулин гларгин приводит к улучшению общего контроля уровня глюкозы, что отражается в снижении уровня глюкозы в крови натощак и уменьшении числа ночных гипогликемий. Гларгин начинает действовать через 1–2 ч после введения. Длительность действия составляет около 24 ч.

В 2015 г. был выпущен инсулин гларгин в концентрации 300 ЕД/мл с увеличенной продолжительностью действия — до 32 ч, что связано с более компактным подкожным депо, чем при введении в дозе 100 ЕД/мл.

Апостериорный метаанализ трех исследований EDITION включил пациентов с СД1, у которых сравнивали параметры эффективности и безопасности инсулина гларгин 100 ЕД/мл с 300 ЕД/мл. Согласно полученным результатам, препараты обладают сходной сахароснижающей эффективностью, но применение 300 ЕД сопровождалось более низким риском тяжелой гипогликемии, особенно в течение первых 8 нед после начала терапии [18]. Однако, как и в случае с инсулином аспарт, существует риск необъективности подобной оценки, что требует изучения профиля безопасности и эффективности препарата в рамках исследований реальной клинической практики.

Инсулин детемир

Инсулин детемир стал вторым аналогом инсулина длительного действия. В молекуле инсулина детемир удалена аминокислота B30, а 14-углеродная алифатическая жирная кислота ацилирована до аминокислоты B29. Это изменение обеспечивает обратимое связывание между альбумином и добавленной жирной кислотой, тем самым замедляя абсорбцию детемира в периферических тканях, поскольку только свободный аналог связывается с рецептором инсулина.

Фармакокинетический профиль инсулина детемир характеризуется пиковой активностью через 6–8 ч после введения и продолжительностью действия до 24 ч. Как и гларгин, детемир вызывает меньшее число эпизодов гипогликемии по сравнению с НПХ. Учитывая сравнительно короткую продолжительность действия, детемир часто вводят дважды в день для обеспечения устойчивого покрытия потребности в базальном инсулине [15].

Инсулин деглудек

В 2012 г. появился новый аналог инсулина сверхдлительного действия, известный как деглудек, который отличается от нативной молекулы отсутствием треонина В-цепи в положении 30 и наличием 16-углеродной жирной двухосновной кислоты (гексадеценовой) у лизина В-цепи в положении 29 посредством ацилирования глутамата. Эти модификации позволяют образовывать мультигексамеры и депо-комплексы в подкожном слое, что способствует медленному высвобождению в системный кровоток [19]. Инсулин деглудек доступен в концентрациях 100 и 200 ЕД/мл, продолжительность действия составляет до 42 ч (период полувыведения 25 ч) [20]. Пролонгированное действие этого аналога человеческого инсулина позволяет более гибко подходить к времени введения препарата, но ограничивает/осложняет его применение из-за необходимости подбора дозы [21].

В 2022 г. были обнародованы результаты исследования InRange (сравнение фармакокинетических и фармакодинамических профилей инсулина гларгин и инсулина деглудек), согласно которым препараты не отличались по своему влиянию на параметры вариабельности гликемии у пациентов с СД1 [20].

Прямые сравнения между инсулином гларгин 300 ЕД/мл и инсулином деглудек проводились также у пациентов с СД2, что сопровождалось сходными показателями динамики гликемического контроля. При этом в исследовании BRIGHT показано, что инсулин гларгин 300 ЕД/мл вызывает меньший риск гипогликемии в течение периода подбора дозы, тогда как в исследовании CONCLUDE столкнулись с техническими проблемами, которые затруднили интерпретацию его результатов [22][23].

Инсулин айкодек (икодек) и BIF — недельные инсулины

Икодек — это относительно новый аналог инсулина, который вводят один раз в неделю, при этом период полувыведения препарата из плазмы составляет 196 ч [24]. В молекуле инсулина икодек заменены 3 аминокислоты, он содержит ацилированную боковой цепью С20-жирную двухосновную кислоту [24]. Молекулярная структура препарата оказалась стабильнее нативной с более длительным периодом полураспада, меньшими сродством к рецепторам, клиренсом и ферментативной деградацией [25].

В настоящее время икодек проходит 3 фазу клинических исследований. Согласно уже опубликованным результатам, икодек в составе базис-болюсной терапии показал сопоставимую или несколько более высокую эффективность в снижении уровня гликированного гемоглобина (HbA1c) без признаков повышенного риска гипогликемии по сравнению с инсулином гларгин у пациентов с СД2 (как инсулин-наивных, так и переведенных с терапии инсулином гларгин 100 Ед/мл) [26].

В настоящее время разрабатываются аналоги базального инсулина еще более длительного действия, в том числе базальный инсулин Fc (BIF), увеличенный период полураспада которого объясняется опосредованной иммуноглобулином FcRn рециркуляцией, тем же процессом, который отвечает за поддержание уровня IgG в сыворотке крови. Предварительные результаты показали пролонгированный сахароснижающий эффект (до 10 дней после однократной инъекции), сравнимый с таковым у обычных базальных инсулинов, без увеличения риска развития гипогликемии [27]. На рис. 3 представлены структуры доступных на сегодняшний день аналогов инсулина и механизм пролонгации их действия.

**Figure:**
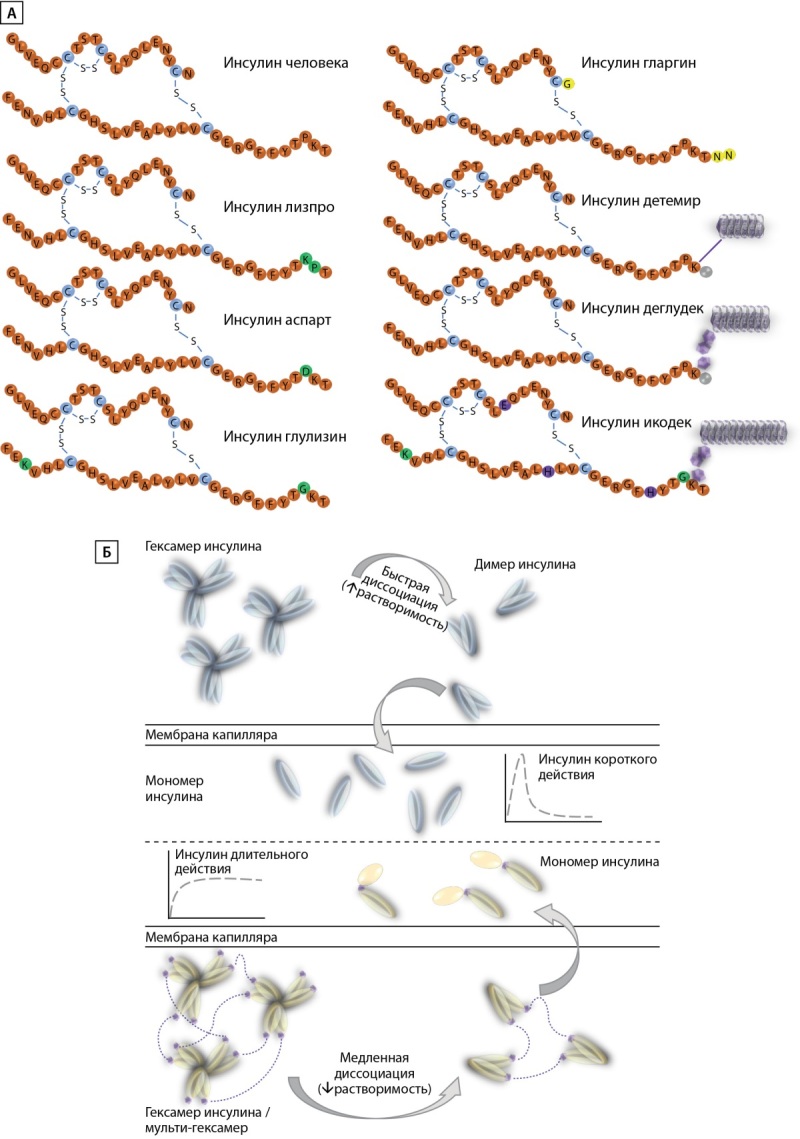
Рисунок 3. Структуры доступных аналогов инсулина (А) и механизм пролонгации их действия (Б).

## Смешанные инсулины

Количество инъекций и простота режима применения имеют решающее значение для значительной части пациентов. Именно поэтому были созданы готовые смеси инсулинов НПХ с инсулинами быстрого действия. В этих препаратах смесь прандиального инсулина с инсулином средней продолжительности действия в одном флаконе или шприц-ручке как обеспечивает снижение гликемии после приема пищи, так и закрывает потребность в базальном инсулине, поскольку фармакокинетика каждого отдельного компонента сохраняется.

Предварительно смешанные инсулины с фиксированным соотношением могут использоваться для повышения приверженности и удобства для пациентов с СД1/СД2, поскольку требуется меньшее количество инъекций по сравнению с традиционным базис-болюсным режимом инсулинотерапии. Фармакокинетический и фармакодинамический профили прандиального компонента в смесях остаются неизменными, поэтому их можно вводить непосредственно перед едой.

Доступны комбинации быстродействующего инсулина лизпро и протамин-инсулина-лизпро, а также быстродействующего инсулина аспарт и протамин-­аспарт-инсулина в различных соотношениях. Следует отметить, что преимущества в отношении гликемического контроля и ночной гипогликемии сохраняются при использовании смесей инсулина лизпро и инсулина аспарт по сравнению с готовыми смесями препаратов человеческого инсулина, которые не обладают этими преимуществами.

Премиксы популярны во многих регионах мира, но в нашей стране не получили широкого распространения.

Фиксированные комбинации базального инсулина и агониста рецептора глюкагоноподобного пептида 1 типа

В настоящее время существуют две фиксированные комбинации базального инсулина с агонистом глюкагоноподобного пептида 1 типа: инсулин гларгин с ликсисенатидом (иГларЛикси) и инсулин деглудек с лираглутидом (иДегЛира).

Согласно имеющейся обширной доказательной базе, фиксированные комбинации агониста рецептора глюкагоноподобного пептида 1 типа и базальный инсулин обладают большей сахароснижающей эффективностью, чем отдельные компоненты, их применение сопровождается меньшим увеличением массы тела и низкой частотой гипогликемии, чем интенсивных режимов инсулинотерапии.

Фиксированные комбинации прамлинтида и инсулина

Еще одним интересным направлением повышения эффективности инсулинотерапии является разработка фиксированных комбинаций прамлинтида с аналогом инсулина. Прамлинтид является аналогом амилина. Подобно эндокринному гормону амилину, оказывает антигипергликемическое действие за счет снижения секреции глюкагона, скорости опорожнения желудка и потребления пищи.

Согласно результатам недавно завершенного сравнительного испытания фазы II (NCT03981627) фиксированной комбинации прамлинтида с быстродействующим аналогом человеческого инсулина A21G (метаболит гларгина), пациентам требовалась меньшая доза инсулина перед приемом пищи, кроме того, пациенты снизили массу тела (в среднем 1,6 кг за 24 дня), в то время как пациенты, получавшие инсулин аспарт, прибавили в среднем 0,4 кг [28]. По всей видимости, это связано со снижением аппетита, которое является одним из физиологических эффектов амилина и, соответственно, прамлинтида.

## Будущее инсулинотерапии

В эру технологического прогресса научные изыскания в области инсулинотерапии идут сразу в нескольких направлениях, ключевыми из которых являются альтернативные способы доставки инсулина и создание новых молекул — «умных» инсулинов.

## Альтернативные способы доставки инсулина

Инъекционный способ введения являлся самым мощным барьером для применения препарата с момента открытия инсулина; по этой причине вскоре после его введения в клиническую практику ученые стали искать альтернативные пути введения [29].

Ингаляционный способ введения

Ингаляционный путь имеет ряд преимуществ перед другими способами доставки препарата. Так, например, общая площадь поверхности легочных альвеол составляет около 90 м², что дает большие возможности для проникновения вдыхаемых лекарств в системный кровоток. В 1925 г. Ганнслен опубликовал результаты применения инсулина, доставляемого в дыхательные пути через стеклянный небулайзер, у 5 пациентов с СД. Однако потребовалось много времени, прежде чем ингаляционный инсулин был одобрен для использования человеком.

В 2006 г. компания Pfizer выпустила Exubera, громоздкую систему для доставки в легкие инсулинсодержащего сухого порошка, которая была снята с производства через несколько лет из-за сохраняющихся опасений по поводу безопасности.

Исследования in vitro показывали, что с помощью Exubera можно доставить по меньшей мере 50% загруженной в него дозы инсулина. При этом высказывались различные опасения, связанные с биодоступностью, которая была значительно меньше, чем при подкожном введении, что, в свою очередь, обуславливает необходимость многократного введения для достижения оптимального эффекта. У 6 из 4740 пациентов, принимавших Exubera на этапе клинических испытаний, был обнаружен рак легких. При этом следует упомянуть, что все эти пациенты ранее курили сигареты, а число зарегистрированных случаев было слишком мало, чтобы можно было сделать вывод о причинно-следственной связи между терапией Exubera и развитием рака легких.

Впоследствии многие другие компании занимались и продолжают заниматься разработкой ингаляционных инсулинов, однако в настоящее время на рынке США доступен только инсулин Afrezza (также известный как инсулин Technosphere), который был одобрен FDA в 2014 г. Afrezza представляет собой инсулин сверхбыстрого действия, предназначенный для контроля постпрандиальной гликемии. Нагруженные инсулином микрочастицы вдыхаются непосредственно в альвеолы и действуют в течение 30 минут. Затем препарат в течение 90 минут выводится из организма. В исследованиях Afrezza сравнивался с инъекционным инсулином лизпро. Согласно полученным данным, частота гипогликемии в группе, принимавшей Afrezza, была значимо ниже [30, 31]. Стойким побочным эффектом ингаляционного инсулина является кашель, поэтому необходимо проведение долгосрочных исследований безопасности и эффективности препарата.

Пероральное применение

Пероральное введение инсулина остается наиболее привлекательной перспективой из-за его удобства, безопасности и физиологического поступления инсулина в портальную циркуляцию. Пероральная доставка инсулина имитирует физиологический (эндогенный) паттерн секреции инсулина за счет увеличения концентрации портального инсулина после абсорбции в кишечнике. В проведенных исследованиях пероральный инсулин также предотвращал аутоиммунное разрушение β-клеток поджелудочной железы [32].

Пероральное введение инсулина менее затратно, так как не требует стерильных условий и сопровождается более высоким уровнем приверженности из-за уменьшения боли и дискомфорта, возникающих при инъекциях.

Однако биодоступность инсулина в значительной степени осложняется физиологическими факторами, такими как изменение рН в желудочно-кишечном тракте (кислый рН в желудке сменяется щелочным рН в кишечнике), ферментативное расщепление и метаболизм в печени. Для преодоления этих ограничений разработаны два основных подхода. Во-первых, это защита от деградации в кислой среде желудка, что позволит препарату всасываться через эпителий кишечника. Подобное протективное действие может обеспечиваться с помощью микропластырей, заключенных в кислотоустойчивые капсулы. Как только капсула достигает просвета кишечника, покрытие растворяется, частицы высвобождаются и прикрепляются к слизистой оболочке, после чего всасывание инсулина происходит через эпителий. Этот подход был реализован в ORMD-0801, рекомбинантном человеческом инсулине для перорального введения, доставляемом пероральной полипептидной системой, содержащей вспомогательные вещества, которые способствуют всасыванию путем ингибирования протеолиза в тонком кишечнике и усиления транслокации пептидов через эпителиальный слой кишечника [33].

Клинические исследования перорального инсулина ORMD-0801

ORMD-0801 содержит рекомбинантный человеческий инсулин. В пилотном исследовании применения ORMD-0801 (8 мг инсулина) в течение 10 дней препарат показал потенциальную возможность его использования у пациентов с СД1.

Чуть позже Элдор и соавт. завершили крупнейшее (на данный момент) плацебо-контролируемое исследование перорального инсулина II фазы, в котором ORMD-0801, содержащий 16 или 24 мг инсулина, добавляли к метформину в терапии пациентов с СД2 в течение 28 дней. Ученые обнаружили, что ночное введение ORMD-0801 предотвращало повышение уровня глюкозы в крови в течение ночи [34]. Препарат также уменьшал 24-часовую гликемию и снижал уровень HbA1c, при этом побочные эффекты были аналогичны таковым в группе плацебо [35]. Несмотря на многообещающие результаты, в последующем эффективность препарата не подтвердилась.

В пероральном инсулине Capsulin в качестве способа доставки используется капсула со специальным покрытием для защиты человеческого инсулина во время его прохождения через желудок, которая затем быстро растворяется в тонкой кишке, в результате чего инсулин контактирует со стенкой кишечника. Второй подход основан на технологиях, разработанных для облегчения всасывания инсулина через слизистую оболочку желудка.

Пероральный инсулин 338 (I338) представляет собой аналог базального инсулина длительного действия, в состав которого входит усиливающий абсорбцию капрат натрия. Инсулин 338 был модифицирован с помощью аминокислотных замен для снижения восприимчивости к протеолитическому расщеплению в ЖКТ. В то же время капрат натрия способствует всасыванию, модулируя плотное соединение желудочного эпителия и повышая текучесть апикальной мембраны.

Клинические исследования I338

Эффективность перорального инсулина 338 в сочетании с капратом натрия была изучена в рандомизированном двойном слепом параллельном исследовании фазы II с активным контролем длительностью 8 нед. В данной работе в качестве препарата сравнения использовался «золотой стандарт» базальной инсулинотерапии — инсулин гларгин 100 ЕД/мл.

В исследование включались инсулин-наивные пациенты с СД2, не достигшие цели на терапии метформином в сочетании или без сочетания с другими пероральными противодиабетическими препаратами. Нежелательные реакции, включавшие преимущественно диарею и назофарингит, были обнаружены у 60% пациентов, получавших I338, и у 68% пациентов, получавших инсулин гларгин. При этом, согласно полученным результатам, между исследуемыми опциями не было выявлено различий по эффективности снижения гликемии. Однако проект был прекращен, поскольку крупномасштабное производство I338 оказалось финансово невыгодным [36].

Недавно Ган и соавт. разработали чувствительные к глюкозе полимерсомы (PMS) с использованием PEG (полиэтиленгликоль)-поли (лактид-со-гликолид) и PMS, нацеленные на ганглиозид-монозиаловую кислоту (GM-1) с пептидом (PEP-PMS) для имитации секреции эндогенного инсулина в ответ на глюкозу посредством GM1-опосредованного трансцитоза [37].

Эти чувствительные к глюкозе PEP-PMS могут высвобождать инсулин в условиях гипергликемии в ответ на GOx-индуцированный H2O2. Исследования in vivo на крысах с диабетом показали, что PEP-PMS проникают через эпителий кишечника посредством GM1-опосредованного эндоцитоза и, наконец, накапливаются в печени. Примечательно, что PEP-PMS достигали высокой концентрации портального инсулина в сыворотке, примерно в 20,3 раза выше по сравнению с нецелевыми PMS. Таким образом, эти нацеленные на печень и чувствительные к глюкозе наноносители рассматривались как многообещающая стратегия пероральной доставки инсулина (NOIDS) для преодоления многочисленных барьеров и стимуляции секреции эндогенного инсулина в печени.

Самый последний подход представлен проглатываемым, самоориентирующимся аппликатором миллиметрового размера (SOMA), который автономно вставляет загруженные лекарством микроштифты в слизистую оболочку желудка [38]. Система содержит сжатую пружину, защищенную сахарным диском, с крошечной иглой, содержащей сублимированный инсулин. После проглатывания SOMA способна к быстрой самонавигации и достигает стабильной точки, когда игла упирается в слизистую оболочку желудка. Затем сахарный диск растворяется, высвобождая пружину и вводя инсулиновую иглу в стенку желудка. Затем отработанное устройство SOMA выводится естественным путем.

Пероральное введение инсулина само по себе не имеет побочных эффектов, но важно тщательно выбирать химические или биологические вспомогательные вещества, которые можно использовать для улучшения поглощения инсулина эпителиальными клетками. Особое значение играет стоимость производства и внедрения новых лекарственных препаратов, содержащих человеческий инсулин или его синтетические аналоги. В связи с вышеперечисленными причинами многие фармацевтические фирмы отказываются от производства пероральных инсулинов на начальных стадиях разработки [39].

Инсулиновая помпа

Введение инсулина с помощью помпы восстанавливает физиологический порто-системный градиент инсулина, предупреждая хроническую системную гиперинсулинемию. В недавней работе были представлены результаты имплантации внутрибрюшинных инсулиновых помп животным и их пополнения с помощью самоориентирующихся таблеток для перорального приема. Таблетки, содержащие концентрированный инсулин, после приема внутрь проходят через магнитную систему ориентации и прикрепляются к стенке кишечника, где расположена инсулиновая помпа, позволяя пополнять резервуар инсулина путем трансмуральной пункции. Встроенная батарея устройства может заряжаться внешними электромагнитными волнами [40].

Интраназальный способ доставки инсулина

Интраназальная доставка давно рассматривается в качестве возможного пути введения инсулина. В том числе многочисленные исследования были посвящены изучению потенциальных носителей и усилителей для интраназальной доставки инсулина. Проникающие в клетку пептиды относятся к категории способных повышать проницаемость через клеточные мембраны. Влияние различных проникающих в клетку пептидов на назальное всасывание инсулина было оценено в исследованиях на животных. Было замечено, что инсулин, вводимый вместе с тестируемыми пептидами (+L-R8, +D-R8, +D-пенетратин и +L-пенетратин), всасывается значительно быстрее и в большем количестве. В частности, снижение уровня глюкозы в крови после совместного введения +D-пенетратина и +L-пенетратина с инсулином составило 30 и 50% соответственно по сравнению с R8. PenetraMax и L-пенетратин не вызывают изменений в слизистой оболочке носа. Авторы исследования пришли к выводу, что L-пенетратин может быть эффективным усилителем проникновения инсулина через слизистую оболочку носа, и эти данные предполагают потенциальное использование интраназального пути для развития инсулинотерапии.

Трансбуккальный способ доставки инсулина

Другим перспективным направлением инсулинотерапии является использование трансбуккального пути введения. Этот путь имеет те же преимущества, что и назальный, с точки зрения малой инвазивности. Ротовая полость, по сути, представляет собой слизистую с большой площадью и высокой васкуляризацией, что позволяет лекарственным препаратам напрямую проникать в системный кровоток [41], что обеспечивает быстрое начало действия.

Однако к существенным недостаткам можно отнести дискомфорт и раздражение слизистой, что провоцирует проглатывание препарата. Несмотря на это, буккальное введение лекарств, особенно белков, является перспективным из-за минимальной инвазивности по сравнению с многократными инъекциями.

Трансдермальный способ доставки инсулина

Трансдермальный способ введения также считается малоинвазивным и потенциально может быть использован для доставки инсулина, поскольку пластыри для чрескожного введения других лекарственных препаратов успешно используются несколько десятков лет. В настоящее время ведутся экспериментальные исследования широкого спектра различных усилителей, способствующих транспорту инсулина через кожу, без значительного ущерба биодоступности и эффективности инсулина.

Вагинальный и ректальный способы доставки инсулина

Вагинальный способ доставки инсулина — еще один потенциальный путь доставки лекарств. Вагина имеет большую площадь слизистой с обилием кровеносных сосудов, которые могут помочь в системной доставке терапевтических агентов.

Эта возможность была подтверждена анализом ex vivo, который показал, что инсулин способен проникать через вагинальную мембрану свиньи на глубину 50–55 мкм. Поэтому были исследованы инкапсулированные наночастицы аскорбата инсулин-хитозан (Ma/Su/GeB), в частности для оценки способности лиофилизированного цилиндра высвобождать нагруженные инсулином наночастицы в слизистую оболочку влагалища. Авторы пришли к выводу, что система лиофилизации, состоящая из Ma/Su/GeB, является подходящим вариантом для доставки пептидов в организм [42].

Инсулин-хитозановый гель in vivo с добавлением двух других усиливающих агентов, таурохолата (ТАУ) и диметил-β-циклодекстрина (ДМ-βЦД), вводили во влагалище и прямую кишку крыс. Концентрация глюкозы в крови снижалась в большей степени у крыс, которым вводили гель DMβCD-хитозан как вагинально, так и ректально, что подтверждает возможность применения этого способа доставки инсулина в организм.

Основываясь на успехе контролируемого высвобождения инсулина, было проведено исследование препаратов Span 40 и Span 60 на крысах с диабетом. Через 1,5 ч после вагинального введения инсулина наблюдалось максимальное снижение уровня глюкозы в крови с сохранением устойчивого и длительного гипогликемического эффекта.

Прямая кишка взрослого человека имеет длину 12–15 см, среднюю площадь поверхности 200–400 см² и рН от 7,2 до 7,4. Ректальное введение инсулина может быть эффективным, поскольку порто-системное шунтирование и лимфодренаж прямой кишки играют значительную роль в системной абсорбции липофильных препаратов. При этом способе введения большое значение имеют высокая скорость и полнота всасывания препарата в системный кровоток [43][44]. Этот путь имеет значительное преимущество, поскольку позволяет избежать эффекта первого прохождения препарата через печень. Кроме того, вводимое лекарство не разлагается под воздействием агрессивной среды и ферментов желудочно-кишечного тракта. Однако недостатком этого пути является наличие слизистого барьера, защищающего эпителиальную стенку [43].

Эту проблему можно решить, разработав энхансер, который будет способствовать проникновению инсулина через защитный барьер. Новый сополимерный гидрогель полиакрилата метакриловой кислоты-со-­гидроксиэтилметакрилата-со-метилакрилата (МАА-со-ГЕМА-со-МА) был приготовлен и растворен в метилцеллюлозе (МЦ) для доставки инсулина в виде ректального суппозитория. Уровень гликемии у крыс с диабетом, которым вводили бинарный гидрогель, содержащий инсулин, свидетельствовал о значительном снижении уровня глюкозы в крови до 7,8 ммоль/л (эффект сохранялся спустя 8,5 ч) по сравнению с подкожным введением инсулина, что приводило к снижению на 10 ммоль/л в течение 4,5 ч [44].

Концентрации инсулина при введении ректально варьируют от 5 до 20 МЕ/кг в течение 24–48 ч с эффективностью высвобождения 75,9%. Кроме того, переносчиком инсулина при данном способе доставки могут быть множественные эмульсии эйкозапентаеновой, олеиновой или докозагексаеновой кислот. При ректальной доставке докозагексаеновая кислота улучшает проницаемость инсулина, действуя как усилитель.

Способы доставки инсулина с использованием микроигл

Микронидлинг — малоинвазивный метод, который можно применять для введения инсулина. Микроиглы сконструированы таким образом, что позволяют им проникать через роговой слой кожи для быстрого высвобождения лекарств, не вызывая необратимого повреждения кожи. В настоящее время на этапе доклинических исследований микроиглы подразделяются на несколько категорий в зависимости от типа и материалов, из которых они сделаны. Так, например, существуют твердые микроиглы для создания отверстия, достаточного для доставки небольших молекул и белков, в том числе и инсулина.

Наилучшая механическая стабильность была достигнута с микроиглами длиной 600 мкм по сравнению с 700 и 800 мкм. Процент проникновения игл длиной 600 мкм оставался на уровне 90% даже после многократных инъекций. Что касается микроигл диаметром 700 и 800 мкм, количество успешных попыток сократилось до менее чем 20%. Возможно, это связано с недостаточными механическими свойствами из-за большей длинны.

Исследование абсорбции in vivo у крыс, предварительно получавших инъекции инсулина через микроиглы, показало снижение уровня глюкозы в крови до 29% от исходных 100% через 5 ч [45]. Альтернативой является использование растворяющихся микроигл, когда лекарство инкапсулируется в растворимую матрицу при введении в кожу. Так, при использовании гиалуроновой кислоты подобные микроиглы полностью растворялись через 1 ч после применения.

В биоразлагаемых микроиглах высвобождение лекарств из матрицы контролируется и может поддерживаться в течение длительного периода. Для стабильного гипогликемического эффекта в матрице подобных игл используется инсулин высоких концентраций, что обусловлено длительностью процесса перехода молекулы из депо в системный кровоток, что может считаться как преимуществом метода, так и его ограничением. Таким образом, биоразлагаемый микронидлинг является потенциальным способом лечения СД с лучшим результатом в поддержании уровня инсулина в сыворотке крови по сравнению с подкожным введением [46].

Основные альтернативные способы доставки инсулина суммированы на рисунке 4 [47].

**Figure:**
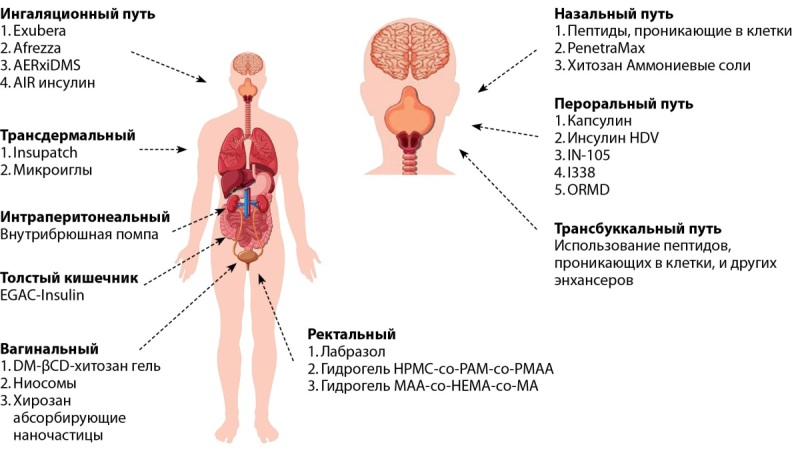
Рисунок 4. Альтернативные способы доставки инсулина

«Умные» инсулины

С 1970-х гг. ученые пытаются создать «чувствительные к глюкозе» препараты инсулина, в связи с чем было подано и одобрено множество патентных заявок, однако ни одна из этих разработок еще не была одобрена для клинического применения. В большинстве опубликованных работ использовали инкапсуляцию инсулина в полимеры, хранящиеся в подкожных депо, и какой-либо механизм для стимуляции ответа на увеличение уровня глюкозы в крови. Однако эти системы обычно слишком медленно реагируют на изменение уровня глюкозы, что делает их непригодными для применения в клинических условиях (табл. 1).

**Table:** Таблица 1. Инсулины с глюкозозависимым механизмом действия [26]

	Препарат	Состав, преимущества и недостатки
«Умные инсулины»Системы на основе полимеров	Глюкозосвязывающие белки	Инсулин, инкапсулированный в глюкозозависимую везикулу/гидрогель
Оксидаза глюкозы	Первые глюкозозависимые инсулины; могут быть токсичны
Фенилбороновая кислота	Катализирует окисление глюкозы в глюконовую кислоту, pH- и триггер-зависимое высвобождение инсулина. Образует обратимые связи с диол-содержащими молекулами (например, глюкозой); чувствительна к рН и концентрации глюкозы
Инсулины с глюкозозависимым действием	Инсулин, ковалентно «пришитый» к фенилбороновой кислоте	Прямая модификация молекулы инсулина для обеспечения глюкозозависимого механизма действия

В 2010 г. было обнаружено, что биоактивность олигосахаридных инсулинов зависит от уровня глюкозы в крови. Однако клинические испытания фазы I не дали убедительных результатов, и программу прекратили. Тем не менее эта концепция продолжает активно развиваться [48][29].

Инсулины с печеночным механизмом действия

Инсулин, секретируемый поджелудочной железой, вначале через воротную вену попадает в печень, а затем распространяется по всему организму, поэтому подкожное введение инсулина приводит к гораздо большему его воздействию на мышцы и меньшему на печень, что обуславливает существенную разницу в эффектах (сначала их очередности, а затем и выраженности) эндо- и экзогенном.

Гепатопреференциальный инсулин был предложен в качестве альтернативы для восстановления физиологического соотношения портального и периферического инсулина, а также для снижения риска гипогликемии и минимизации увеличения массы тела. Инсулин пэглизпро представляет собой молекулу, состоящую из цепи полиэтиленгликоля, ковалентно связанной с лизином B28 инсулина лизпро, и оказывает гепатопреференциальное действие благодаря своему большому гидродинамическому размеру [49].

Более 6000 пациентов с СД1 и СД2 были включены в программу клинических испытаний IMAGINE фазы 3, в которой инсулин пэглизпро неизменно демонстрировал большее снижение HbA1c, меньшую вариабельность гликемии, снижение ночных гипогликемий и тенденцию к меньшему набору веса по сравнению с инсулинами гларгином или НПХ [49].

Несмотря на свою потенциально более высокую эффективность, инсулин пэглизпро связан с увеличением содержания жира в печени и триглицеридов, а также с более высокой частотой повышения уровня аминотрансфераз. Хотя это не было связано с тяжелым поражением печени, производитель решил остановить программу разработки в 2015 г. [49–51].

Другой интересной разработкой является пероральный гепатоцит-ориентированный везикулярный инсулин на основе липидных наночастиц. Клинические испытания фазы II/III были завершены в 2009 г. Согласно полученным результатам, на фоне терапии новым типом инсулина отмечалось значительное снижение средней площади постпрандиальной глюкозы в плазме под кривой (AUC) по сравнению с плацебо без увеличения каких-либо рисков, связанных с безопасностью. Однако при повышении концентрации инсулина ожидаемая дозозависимая реакция отсутствовала. С тех пор компания изменила назначение препарата для использования в инъекциях и инфузиях и добилась хороших результатов в гликемическом контроле у пациентов с СД1 в клиническом исследовании фазы IIb NCT02794155 [14].

## ДОСТУПНОСТЬ ИНСУЛИНОВ

Существенными проблемами, которые невозможно игнорировать, являются цена инновационных инсулинов и доступ к этой жизненно важной терапии в целом. Инсулин часто находится в крайне ограниченном количестве и стоит непомерно дорого в развивающихся странах, но даже в странах с сильной экономикой доступ к инсулину остается проблематичным [5].

Эта ситуация примечательна, учитывая, что первоначальный патент на инсулин был продан за 1 доллар Университету Торонто, поскольку было сочтено неэтичным получать прибыль от открытия, которое могло бы спасти жизни. Знаменитое заявление Бантинга: «Инсулин принадлежит не мне, он принадлежит миру» [4].

За столетие, прошедшее с момента заявления Бантинга, старые инсулины были заменены постоянно совершенствующимися продуктами, защищенными новыми патентами, что привело к почти экспоненциальному росту стоимости новых препаратов на свободном рынке. Высокая стоимость современных препаратов инсулина существенно ограничивает возможности их закупки для обеспечения нужд пациентов. Так, в 2020 г. в РФ только 50,6% пациентов СД2 получали современные аналоги инсулина, что, несомненно, является недостаточным и представляет собой проблему, требующую глобального решения [49][52].

## ЗАКЛЮЧЕНИЕ

В 2021 г. исполнилось 100 лет со дня открытия инсулина, события, навсегда изменившего жизнь людей с СД. Миллионы людей во всем мире ежедневно сталкиваются с чудом инсулинотерапии. Болезнь, от которой в 1920 г. дети и подростки умирали в течение 2 лет, превратилась в болезнь, с которой люди могли справиться и прожить долгую продуктивную жизнь.

За последнее столетие инсулинотерапия шагнула далеко вперед, что существенно улучшило качество жизни наших пациентов. Но исследования активно продолжаются, в том числе в области альтернативных способов доставки инсулина, которые являются более удобными для пациента, а также в области разработок «умных» молекул, которые будут обладать глюкозозависимым действием.

## ДОПОЛНИТЕЛЬНАЯ ИНФОРМАЦИЯ

Источники финансирования. Работа поддержана грантом РНФ (проект №20-75-10013).

Конфликт интересов. Авторы декларируют отсутствие явных и потенциальных конфликтов интересов, связанных с содержанием настоящей статьи.

Участие авторов. Куркин Д.В., Колосов Ю.А., Робертус А.И. — разработка концепции и научное консультирование; Бакулин Д.А., Робертус А.И., Иванова О.В, Кудрин Р.А., Крысанов И.С., Макарова Е.В., Морковин Е.И., Стрыгин А.В., Горбунова Ю.В., Макаренко И.Е., Драй Р.В., Павлова Е.В. — сбор материала, составление таблиц, подбор рисунков, написание текста статьи.

Все авторы одобрили финальную версию статьи перед публикацией, выразили согласие нести ответственность за все аспекты работы, подразумевающую надлежащее изучение и решение вопросов, связанных с точностью или добросовестностью любой части работы.
